# Supplementary material for: DNA polymerase I is an efficient reverse transcriptase that mediates RNA-templated DNA repair synthesis
Source: bioRxiv. 2025 Dec 23:2025.12.22.696026. Preprint. [Version 1] doi: 10.64898/2025.12.22.696026 (PMC12776460; doi:10.64898/2025.12.22.696026)
Supplement: Supplement 1 [file media-1.pdf]

**Supplemental Information**

# DNA polymerase I is an efficient reverse transcriptase that mediates RNA-templated DNA repair synthesis

Frances C. Lowder, Abigail H. Kendal, and Lyle A. Simmons*

Department of Molecular, Cellular, and Developmental Biology, University of Michigan, Ann Arbor, MI 48109

*LAS: Department of Molecular, Cellular, and Developmental Biology, University of Michigan, Ann Arbor, Michigan 48109-1055, United States. Phone: (734) 763-7142, Fax: (734) 647-0884 E-mail: lasimm@umich.edu

Short title:

**Keywords:** Reverse transcriptase, *Bacillus subtil*is, RNA-DNA hybrid, genome instability, DNA polymerase I

**Supplemental Table 1.** Bacterial Pol Is with reverse transcriptase activity

| Organism | Gram-stain | D | E | D | Y-H | D | Active 3′-5′ Exonuclease? | Notes |
|---|---|---|---|---|---|---|---|---|
| *Bacillus caldolyticus* EA.1[1] | Positive | - | E | - | H | - | No | |
| *Bacillus subtilis* | Positive | - | E | - | H | - | No | First studied in this work. |
| *Caldibacillus cellulovorans* CompA.2[1] | Positive | - | E | - | Y | - | No | |
| *Caldicellulosiruptor saccharolyticus* Rt69B.1[1] | Positive | - | - | - | - | - | No | |
| *Caldicellulosiruptor saccharolyticus* Tok13B.1[1] | Positive | - | - | - | - | - | No | |
| *Caldicellulosiruptor saccharolyticus* Tok7B.1[1] | Positive | - | - | - | - | - | No | |
| *Dictyoglomus thermophilum* Rt46B.1[1] | Negative | - | - | D | H | - | No | |
| *Escherichia coli*[2–8] | Negative | D | E | D | Y | D | Yes | |
| *Geobacillus stearothermophilus*[9–11] | Positive | - | E | - | H | - | Yes | |
| *Listeria monocytogenes* | Positive | - | E | - | H | - | Yes | First studied in this work. |
| *Micrococcus luteus*[3] | Positive | - | - | - | - | - | No | |
| *Mycolicibacterium smegmatis*[12] | - | - | - | - | - | - | No | Bound 1 $Mn^{2+}$ using non-conserved residues[12] |
| *Pseudomonas aeruginosa*[7] | Negative | D | E | D | Y | D | Yes | |
| *Shigella sonnei*[7] | Negative | D | E | D | Y | D | Yes | |
| *Staphylococcus aureus*[7] | Positive | - | E | - | Y | - | No | |
| *Streptococcus agalactiae*[13] | Positive | - | E | - | H | - | No | |
| *Streptomyces coelicolor*[14] | Positive | - | - | - | - | - | No | |
| *Thermoactinomyces vulgaris*[9] | Positive | - | E | - | H | - | No | |
| *Clostridium thermosulfurogenes*[1] | Positive | - | - | - | Y | - | No | |
| *Thermoclostridium stercorarium*[1] | Positive | - | - | - | Y | - | No | |
| *Thermotoga neapolitana*[1,15] | Negative | D | E | D | Y | D | Yes | Modified to lose 3′-5′[1] |
| *Thermus aquaticus*[1,16,17] | Negative | - | - | - | - | - | No | |
| *Thermus filiformis*[1] | Negative | - | - | - | - | - | No | |
| *Thermus thermophilus*[1,18,19] | Negative | - | - | - | - | - | No | Activity studied with $Mn^{2+}$[18,19] |
| *Vibrio cholerae* non-O1[7] | Negative | D | E | D | Y | D | Yes | |

Note that the identity of the *E. coli* hybrid polymerase purified by Lee-Huang and Cavalieri is unclear[6], but the reference is included in Sup. Table 1 since other works studying *Ec*Pol I make reference to it[4].

**Supplemental Table 2.** *B. subtilis* strains used in this study.

| Strain Identifier | Relevant Genotype | Citation |
|---|---|---|
| FCL3 | Δ*polA* | JWS235[20] |
| FCL10 | native PY79 | Youngman |
| FCL11 | Δ*rnhC* | JRR48[20] |
| FCL12 | Δ*rnhC*, Δ*polA* | JRR64[20] |

**Supplemental Table 3.** Oligonucleotides used in this study.

| Oligonucleotide | Purpose | Sequence (5′-3′) |
|---|---|---|
| oJR46 | Amplifying pE-SUMO vector (F) | TCGAGCACCACCACCACCACCACTGAG |
| oJR47 | Amplifying pE-SUMO vector (R) | ACCTCCAATCTGTTCGCGGTGAGCCTCAATAATATCG |
| prFCL120 | Amplifying *SapolA* (F) | ctcaccgcgaacagattggaggtGTGAATAAATTAGTATTAATCGATGG |
| prFCL121 | Amplifying *SapolA* (R) | gtggtggtggtggtgctcgaTTATTTTGCATCATACCAGGTTGCACC |
| prFCL122 | Amplifying Ec*polA* (F) | ctcaccgcgaacagattggaggtATGGTTCAGATCCCCCAAAATCCACTTATCC |
| prFCL123 | Amplifying Ec*polA* (R) | gtggtggtggtggtgctcgaTTAGTGCGCCTGATCCCAGTTTTCG |
| prFCL124 | Amplifying pTwist Insert (F) | GGCTCACCGCGAACAGATTGGAGGT |
| prFCL125 | Amplifying pTwist Insert (R) | CAGTGGTGGTGGTGGTGGTGCTCGA |
| oFCL7 | Assay – DNA Template | GCA*A*T*CGACTCGTAAGCATGGTTCACTACTCGCTGCTTGATGCTCAATCG |
| oFCL8 | Assay – DNA Primer | /5IRD800/C*G*A*TTGAGCATCAAGCAGCG |
| oFCL16 | Assay – Short RNA Primer | /5IRD700/G*C*A*GAGCTAGC |
| oFCL18 | Assay – Long RNA Primer | /5IRD700/A*C*A*GCGTTCCCT |
| oFCL20 | Assay – Short RNA Ladder | /5IRD700/GCAGAGCTAGCTTACGATCG |
| oFCL21 | Assay – Hybrid Primer | /5IRD700/C*A*A*GTCATCAAATGG |
| oFCL22 | Assay – Hybrid DNA Template | TGAGTAAGTTGGTATCCGAGGTACTATGAGCTTCTGGACCATTTGATGACTTG |
| oFCL23 | Assay – Hybrid 1 nt Template | TGAGTAAGTTGGTATCCGAGGTACTATGAGCTUCTGGACCATTTGATGACTTG |
| oFCL24 | Assay – Hybrid 5 nt Template | TGAGTAAGTTGGTATCCGAGGTACTATGAGCUUCTGGACCATTTGATGACTTG |
| oFCL25 | Assay – Hybrid 10 nt Template | TGAGTAAGTTGGTATCCGAGGTACUAUGAGCUUCTGGACCATTTGATGACTTG |
| oFCL26 | Assay – Hybrid 15 nt Template | TGAGTAAGTTGGTATCCGAGGUACUAUGAGCUUCTGGACCATTTGATGACTTG |
| oFCL27 | Assay – DNA Ladder | /5IRD800/C*G*A*TTGAGCATCAAGCAGCGAGTAGTGAACCATGCTTACGAGTCGATTGC |
| oJR227 | Assay – Short RNA Template | /5IRD800CWN/CGAUCGUAAGCUAGCUCUGC |
| oJR336 | Assay – Long RNA Template | /5IRD800CWN/CUGGAGGAUGGAGGAUGGUGGAGAUGUGAGGGAACGCUGU |

* indicates phosphorothioate linkages

IRD indicates an infrared dye at the 5′ end or 3′ end as indicated, 700 or 800 indicates the wavelength of excitation, and CWN indicates a NHS ester conjugation.

Red bases indicate ribonucleotides.

**Supplemental Table 4.** Plasmids used in this study.

| Plasmid Identifier | Vector | Insert |
|---|---|---|
| pJR22 | pE-SUMO | *BspolA*[21] |
| pFCL56 | pE-SUMO | *SapolA* |
| pFCL57 | pTwist-Kan | *LmpolA** |
| pFCL58 | pTwist-Kan | MspolA* |
| pFCL59 | pE-SUMO | *EcpolA* |
| pFCL61 | pE-SUMO | *LmpolA** |
| pFCL62 | pE-SUMO | MspolA* |

***** indicates genes codon optimized for expression in *E. coli*

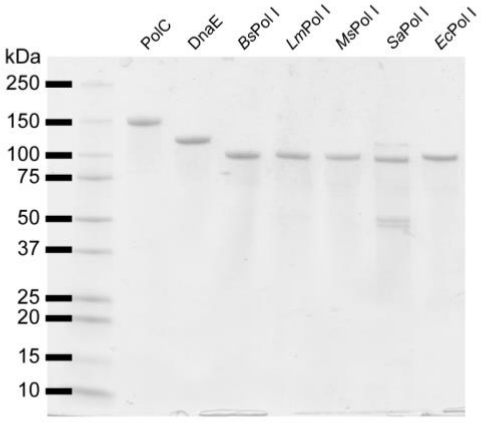


**Sup. Figure 1. Purified proteins used in biochemical assays.** 2 µg of each protein used for extension assays are shown on a Coomassie-stained, SDS-PAGE.

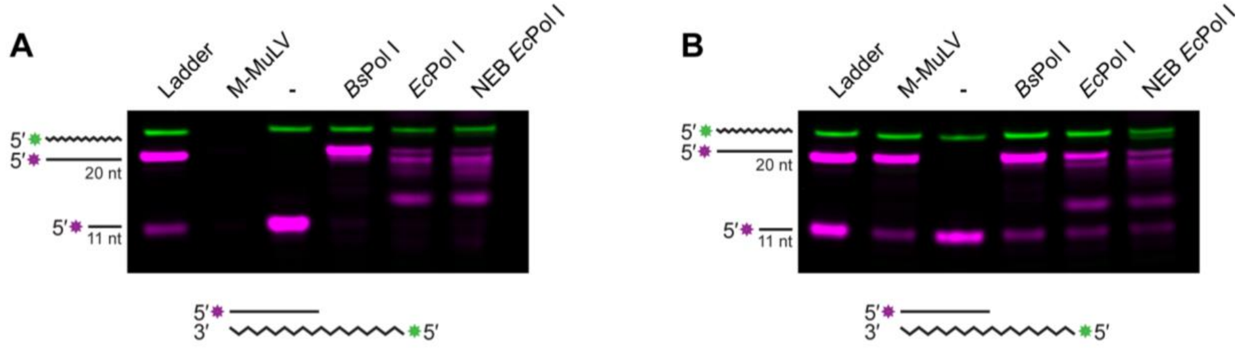


**Sup. Figure 2. Commercial EcPol I has reverse transcriptase activity.** (A) Primer extension in DNA extension buffer using an RNA template. (B) Primer extension from an RNA template in reverse transcriptase buffer.

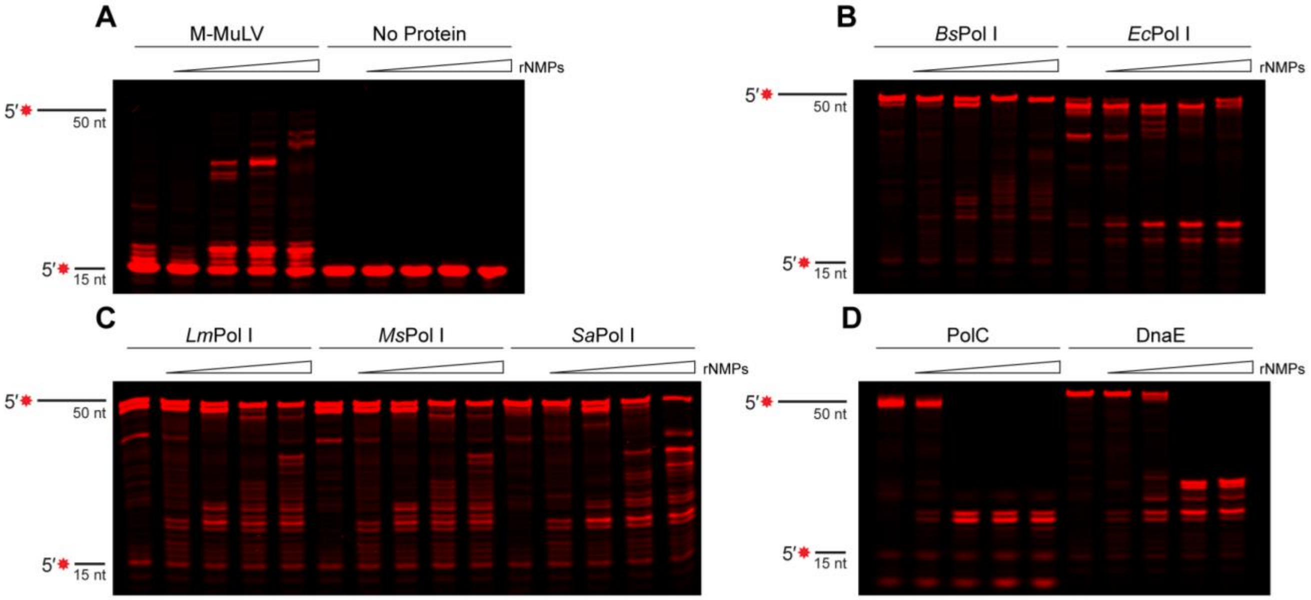


**Sup. Figure 3. Pol I traverses embedded ribonucleotides under DNA extension conditions.** (A-D) Primer extension products generated by the indicated polymerase after 20 minutes using substrates with increasing stretches of ribonucleotides.

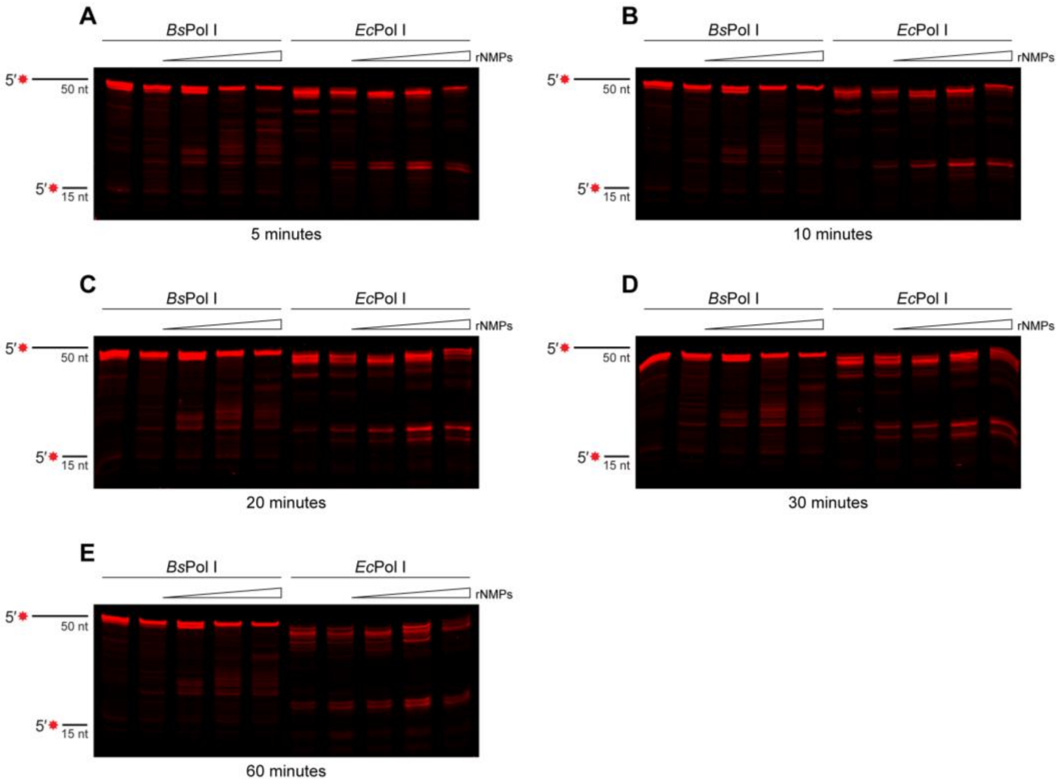


**Sup. Figure 4. EcPol I nuclease activity degrades reaction products over time.** (A-E) Primer extension products generated by the indicated polymerase using substrates with increasing stretches of ribonucleotides. Reaction incubation times are indicated below each gel.

**References:**

1. Shandilya H, Griffiths K, Flynn EK, et al. Thermophilic bacterial DNA polymerases with reverse-transcriptase activity. *Extremophiles*. 2004;8(3):243-251. doi:10.1007/s00792-004-0384-5

2. Karkas JD, Stavrianopoulos JG, Chargaff E. Action of DNA polymerase I of *Escherichia coli* with DNA-RNA hybrids as templates. *Proc Natl Acad Sci*. 1972;69(2):398-402. doi:10.1073/pnas.69.2.398

3. Wells RD, Fluegel RM, Larson JE, Schendel PF, Sweet RW. Comparison of some reactions catalyzed by deoxyribonucleic acid polymerase from avian myeloblastosis virus, *Escherichia coli*, and *Micrococcus luteus*. *Biochemistry*. 1972;11(4):621-629. doi:10.1021/bi00754a025

4. Loeb LA, Tartof KD, Travaglini EC. Copying natural RNAs with *E. coli* DNA polymerase I. *Nat N Biol*. 1973;242(116):66-69. doi:10.1038/newbio242066a0

5. Ricchetti M, Buc H. *E. coli* DNA polymerase I as a reverse transcriptase. *EMBO J*. 1993;12(2):387-396. doi:10.1002/j.1460-2075.1993.tb05670.x

6. Lee-Huang S, Cavalieri LF. Isolation and properties of a nucleic acid hybrid polymerase. *Proc Natl Acad Sci*. 1964;51(6):1022-1028. doi:10.1073/pnas.51.6.1022

7. Harada F, Nakano T, Kohno T, Mohan S, Taniguchi H, Sano K. RNA-dependent DNA polymerase (RT) activity of bacterial DNA polymerases. *Bull Osaka Méd Coll*. 2005;51(1):35-41. doi:10.57371/00000410

8. Chandramouly G, Zhao J, McDevitt S, et al. Polθ reverse transcribes RNA and promotes RNA-templated DNA repair. *Sci Adv*. 2021;7(24):eabf1771. doi:10.1126/sciadv.abf1771

9. Trent G, Robert H James, Fen H. Method of reverse transcription using *Bacillus steraothermophilus* or *Thermoactinomyces vulgaris* DNA polymerase. 2001.

10. Shi C, Shen X, Niu S, Ma C. Innate reverse transcriptase activity of DNA polymerase for isothermal RNA direct detection. *J Am Chem Soc*. 2015;137(43):13804-13806. doi:10.1021/jacs.5b08144

11. Jackson LN, Chim N, Shi C, Chaput JC. Crystal structures of a natural DNA polymerase that functions as an XNA reverse transcriptase. *Nucleic Acids Res*. 2019;47(13):6973-6983. doi:10.1093/nar/gkz513

12. Ghosh S, Goldgur Y, Shuman S. Mycobacterial DNA polymerase I: activities and crystal structures of the POL domain as apoenzyme and in complex with a DNA primer-template and of the full-length FEN/EXO–POL enzyme. *Nucleic Acids Res*. 2020;48(6):3165-3180. doi:10.1093/nar/gkaa075

13. Bahrami F, Jestin JL. *Streptococcus agalactiae* DNA polymerase I is an efficient reverse transcriptase. *Biochimie*. 2008;90(11-12):1796-1799. doi:10.1016/j.biochi.2008.07.006

14. Bao K, Cohen SN. Reverse transcriptase activity innate to DNA polymerase I and DNA topoisomerase I proteins of *Streptomyces* telomere complex. *Proc Natl Acad Sci*. 2004;101(40):14361-14366. doi:10.1073/pnas.0404386101

15. Keith B, W MT, Fred R, Joseph SF, Rachel S, Shawn S. DNA polymerases with improved activity. 2015.

16. Jones MD, Foulkes NS. Reverse transcription of mRNA by *Thermus aquaticus* DNA polymerase. *Nucleic Acids Res*. 1989;17(20):8387-8388. doi:10.1093/nar/17.20.8387

17. Barnes WM, Zhang Z, Kermekchiev MB. A single amino acid change to *Taq* DNA polymerase enables faster PCR, reverse transcription and strand-displacement. *Front Bioeng Biotechnol*. 2021;8:553474. doi:10.3389/fbioe.2020.553474

18. Myers TW, Gelfand DH. Reverse transcription and DNA amplification by a *Thermus thermophilus* DNA polymerase. *Biochemistry*. 1991;30(31):7661-7666. doi:10.1021/bi00245a001

19. Cai D, Behrmann O, Hufert F, Dame G, Urban G. Capacity of r*Tth* polymerase to detect RNA in the presence of various inhibitors. *PLoS ONE*. 2018;13(1):e0190041. doi:10.1371/journal.pone.0190041

20. Randall JR, Nye TM, Wozniak KJ, Simmons LA. RNase HIII is important for Okazaki fragment processing in *Bacillus subtilis*. *J Bacteriol*. 2019;201(7):e00686-e00699. doi:10.1128/jb.00686-18

21. Schroeder JW, Randall JR, Hirst WG, O’Donnell ME, Simmons LA. Mutagenic cost of ribonucleotides in bacterial DNA. *Proc National Acad Sci*. 2017;114(44):11733-11738. doi:10.1073/pnas.1710995114
